# Hair Restorative Testimonial

**Published:** 1875-10

**Authors:** 


Hair Restorative Testimonial.—A Chicago chemist alludes to his new combination for restoring hair in the following manner: A little applied to the inkstand has given it a coat of bristles, making a splendid pen-wiper at little cost. We applied the lather to a ten-penny nail, and the nail is now the handsomest lather-brush you ever saw, with beautiful soft hair growing from the end of it, some five or six feet in length. Applied to door-stones, it does away with the use of the mat; applied to the floor, it will cause to grow therefrom hair sufficient for a Brussels carpet. A little weak lather sprinkled over a barn makes it impervious to the wind, rain, or cold. It is good to put inside of children's cradles, sprinkle on the road-side, or anywhere where luxurious grass is wanted for use or ornament. It produces the effect in ten minutes.—*Pharmacal Gazette.*

